# A New Dental College

**Published:** 1894-07

**Authors:** 


### A New Dental College.

A new dental college was organized about a year ago in Tacoma, State of Washington, and recently brought to a close its first session.

The faculty consists of the leading men of the profession in that city; and from information we have received they have done excellent work during the term just closed. They, of course, labor under embarrassment, incident to a new enterprise and to being so far distant, almost amounting to isolation, from other institutions of a similar character. It is to be hoped, however, that at a very early day these difficulties will all be cleared away, and that an institution as good as the best shall grow out of this beginning.

